# Study on Experimental Parameters of Alkali-Assisted Extraction of Aluminum from Fly Ash

**DOI:** 10.3390/ma18071568

**Published:** 2025-03-30

**Authors:** Bingchao Zhao, Yufeng Guo, Wei Wang, Xin Wan, Shenglin He, Tongxiaoyu Wang

**Affiliations:** 1 College of Energy Engineering, Xi’an University of Science and Technology, Xi’an 710054, China; zhaobc@xust.edu.cn (B.Z.); wangw@xust.edu.cn (W.W.); 23203077039@stu.xust.edu.cn (X.W.); 22103077014@stu.xust.edu.cn (S.H.); 24203226118@stu.xust.edu.cn (T.W.); 2 Key Laboratory of Mine Mining and Disaster Prevention in West China, Ministry of Education, Xi’an University of Science and Technology, Xi’an 710054, China

**Keywords:** coal fly ash, sodium carbonate, activation, solid waste treatment, hydrochloric acid

## Abstract

Extracting aluminum from FA is an effective way to improve its utilization rate. Since aluminum oxide is found in high polymerization degree, inert substances such as mullite and sodium aluminosilicate make the reaction process difficult because of their stable chemical properties; within these highly polymerized matrices, the chemical stability of alumina typically persists across a broad temperature range from 1000 °C to over 1600 °C. To address the issue of stable mullite structure that hinders aluminum extraction, a combined acid-base method using sodium carbonate as an activating agent and hydrochloric acid at a temperature of 100 °C as a leaching agent is employed. XRD and SEM were used to analyze the phase characterization and microstructure of fly ash before and after activation and acid leaching, examining the effects of activation parameters and acid leaching parameters on the activation of FA and the aluminum extraction rate. The research results indicate that after calcination activation, mullite is transformed into zeolite, which is easily soluble in acid, and the aluminum within the activated molten material is transferred to the filtrate through acid leaching, achieving the goal of extracting aluminum. Under the activating conditions with sodium carbonate flux, the Al-O bonds in mullite are broken, the crystal structure is transformed, and the aluminum compounds obtained from hydrochloric acid leaching have a stable form of existence, which has a low impact on the error of the experimental results. When the material ratio of fly ash to sodium carbonate is 1:0.7, after reacting at a calcination temperature of 880 °C for 1.5 h, and leaching in 6 mol/L hydrochloric acid at 100 °C with a solid-liquid ratio of 1:6 for 2 h, the extraction rate of aluminum in fly ash is the highest, reaching 97%.

## 1. Introduction

Fly ash (FA), a principal byproduct of coal combustion in power plants, poses a significant challenge in terms of land resource consumption and has deleterious effects on atmospheric, soil, and aquatic resources [1,2,3]. Its low effective utilization rate, coupled with the presence of toxic and hazardous metals such as arsenic, lead, and cadmium, predisposes it to cause secondary pollution [4]. Currently, the primary method of harnessing fly ash involves the recycling of its raw materials to extract elements that are amenable to subsequent utilization, such as silicon, aluminum, gallium, germanium, iron, yttrium, titanium and vanadium, with aluminum and silicon collectively accounting for 70% to 82% of its total composition [5]. Transforming fly ash into a substrate rich in valuable extractable elements is a pivotal strategy for enhancing its economic value [6].

Aluminum is predominantly present in FA in the form of aluminum oxide, which is primarily distributed within the mullite (3Al_2_O_3_·2SiO_2_) mineral phase and the aluminosilicate (NaAlSiO_4_) glass phase [7,8]. Phase analyses reveal that alumina within aluminosilicate glass phases predominantly exists in amorphous or low-crystallinity states exhibiting enhanced reactivity [9,10]. Under appropriate activation conditions (e.g., thermal treatment >600 °C or chemical modification), these glass phases undergo structural reorganization through depolymerization of Si-O-Al networks, significantly improving alumina accessibility to acidic/alkaline leaching agents. Changing its structure via chemical activation enhances its reactivity at room temperature, reducing the energy consumption and cost of the process. In contrast, the silicoaluminate components within the mullite crystal structure, due to their tight bonding, have high thermal stability. The high polymerization degree results in low reactivity of alumina [9,11]. During aluminum recovery, the alumina in the mullite phase barely reacts with acids or alkalis, leading to low leaching rates under conventional conditions. This seriously restricts aluminum extraction efficiency in coal fly ash resource utilization [12,13]. These mineralogical characteristics necessitate the adoption of more efficient pretreatment or modification techniques in the resource utilization process to promote the liberation of silicon and aluminum constituents. As a result, coal fly ash with abundant mullite needs special pre-treatment or more intense reaction conditions to boost aluminum recovery, adding to the process complexity and cost. Zai-ming Yang [14] utilized limestone sintering of fly ash to extract aluminum oxide, and their research elucidated the impact of varying calcium-to-aluminum ratios on the leaching of aluminum oxide; however, this sintering method operates at high temperatures, significantly increasing energy consumption during the extraction process, and also consumes substantial amounts of non-renewable limestone resources, generating a large quantity of silicon slag, which complicates the reuse of silicon components. Zhen-kai Liang [15] employed CaCl_2_ as a chlorinating agent, capable of disrupting the mullite mineral phase and generating phases susceptible to acid decomposition. This method operates at lower sintering temperatures and consumes less energy, thus eliminating the need for a complex batching system. However, the substantial hydration products of calcium sulfate generated can increase the viscosity of the slurry, enhancing the diffusion resistance of aluminum ions and affecting the leaching efficiency of aluminum. Yan-xia Du [16] adopted a pre-desiliconization alkali-lime sintering method to extract aluminum oxide from fly ash. This method has relatively lower energy consumption and slag production compared to the limestone soda method, but the low efficiency of pre-desiliconization may lead to the high moisture content in the silicon slag, increasing the production costs of calcium silicate and also the cost of clinker sintering. The use of sodium carbonate [17,18,19] as an activating agent for the activation sintering of fly ash induces a reaction in the aluminum oxide present in the mullite (3Al_2_O_3_·2SiO_2_) mineral phase, transforming it into the more acid-soluble nepheline (NaAlSiO_4_). Subsequent acid leaching processes, followed by solid-liquid separation of the leachate, yield a concentrated aluminum ion solution, thereby achieving effective extraction of aluminum from FA. This method, characterized by lower sintering temperatures and the use of hydrochloric acid leaching [20,21], with the ease of recovering aluminum ions from the leachate, has been widely applied in the research on aluminum extraction from fly ash. However, in the leaching process, leaching time and concentration affect the recovery of silica and alumina. For instance, excessive leaching time can decrease aluminum extraction due to the kinetic mechanism shift (from chemical reaction control to diffusion control) and side reaction accumulation.

This study utilizes fly ash from the Hancheng Power Plant as the experimental feedstock, sodium carbonate as the experimental auxiliary, and hydrochloric acid as the experimental leaching agent. A combined acid-base treatment method for fly ash was employed to investigate the extraction rate of aluminum in the aluminum-containing filtrate under various experimental parameter conditions. The effects of calcination temperature, the material ratio of fly ash to sodium carbonate, calcination duration, hydrochloric acid leaching concentration, solid-liquid ratio, and leaching time on the aluminum extraction rate were examined. This study explored the activation mechanism of sodium carbonate on fly ash and analyzed the patterns of aluminum extraction rates. X-ray powder diffractometry (XRD) and thermal field emission scanning electron microscopy (SEM) were employed to characterize the phases of fly ash before and after sodium carbonate activation and acid leaching, as well as to scan the structural changes in the micro-particles.

## 2. Experimental Details

### 2.1. Experimental Samples and Equipment Experimental Methods

The raw material sample selected for this experiment is fly ash from the Hancheng Power Plant in Shaanxi Province, whose composition is detailed in Table 1.

The chemical reagents used in this experiment include anhydrous sodium carbonate (Na_2_CO_3_, AR), sourced from Tianjin Damao Chemical Reagent Factory, Tianjin, China, and hydrochloric acid (HCl, AR), also from Tianjin Damao Chemical Reagent Factory, Tianjin, China. The experimental equipment comprises a CN-YH2003 digital balance manufactured by Kunshan Youkeweit Electronic Technology, (Jiangsu, China) Co., Ltd., a KSL-1200X muffle furnace produced by Hefei Kejing Material Technology, Anhui, China. Co., Ltd., a DF-101S heat-collecting constant temperature magnetic stirrer from Tianjin Huaxin Instrument, (Tianjin, China) Co., Ltd., and a DHG-9015A electric convection oven from Shanghai Yiheng Scientific Instrument, (Shanghai, China) Co., Ltd., among others.

### 2.2. Experimental Methods

#### 2.2.1. Raw FA Treatment

The FA used in this experiment underwent a drying and dehydration treatment. The sample was meticulously retrieved via the quartering technique and subsequently rinsed with deionized water to eliminate surface dust, followed by drying at a temperature of 105 °C in a convection oven for a duration of 6 h to ensure complete moisture elimination The dried fly ash was then sealed and stored for future use.

#### 2.2.2. Activation Process

This study uses a combined acid–alkaline method to extract aluminum from fly ash, referencing and optimizing Elsayed M.’s approach [22]. Sodium carbonate is used as an alkaline activator. After drying as described in Section 2.2.1, the fly ash was uniformly mixed and ground with sodium carbonate in a specific ratio to a powdery form, placed in a ceramic crucible, and then introduced into a muffle furnace for calcination at an elevated temperature for a predetermined duration. The crucible was removed to cool, and the calcined melt was re-ground thoroughly before being placed in a drying oven at 60 °C for further drying.

#### 2.2.3. Acid Leaching

A specific volume of hydrochloric acid with a predetermined concentration was taken in a test tube and placed into a beaker, to which an appropriate amount of deionized water was added. The dried melt was slowly poured into the solution and then transferred to a magnetic stirrer, where it was continuously stirred under isothermal conditions in a water bath for a set period. After the acid leaching process was completed, solid–liquid separation was performed, and the concentration of aluminum in the leachate was determined. The extraction yield of aluminum oxide in the leachate is calculated using Equation (1).

(1) $$\omega_{Al_{2}O_{3}}=\frac{m_{0}(Al_{2}O_{3})}{m(Al_{2}O_{3})}=\frac{2\times V\times 102\times 10^{-3}}{\omega_{0}\times m_{1}}\times 100\%$$

where *m*_0_(*Al_2_O_3_*) represents the mass of aluminum oxide in the acid leachate, in grams (g); *m*(*Al_2_O_3_*) represents the total mass of aluminum oxide in the fused material, in grams (g); *V* represents the volume of hydrochloric acid leaching solution, in milliliters (mL); *ω*_0_ represents the mass fraction of aluminum oxide in the FA, in percent (%); *m*_1_ represents the mass of the FA used in this experiment, in grams (g).

#### 2.2.4. Analytical and Characterization Methods

(i) X-ray powder diffraction (XRD) analysis, commonly used to analyze the crystalline structure of fly ash and determine its crystalline phase composition, is a vital technique for in-depth analysis of inorganic compound crystalline structures. In this study, a Bruker D8 Advance X-ray powder diffractometer (Cu Kα) was used for phase analysis via step-scanning with parameters set at 35 kV tube voltage, 25 mA tube current, 0.05°/s step width, and an angular range of 30–100°. This setup aimed to investigate the impact of the alkaline activation process on the crystalline structure of fly ash by comparing XRD spectra changes before and after calcination to evaluate mineral transformation or decomposition. (ii) A JSM-IT800 thermal field-emission scanning electron microscope (SEM) was selected to observe the micro-morphology of fly ash samples, revealing their particle shape, size, and surface characteristics. It also examined the effects of calcination activation and acid leaching on the microstructure of fly ash, such as particle etching, dissolution, and surface morphology changes under initial alkaline conditions. G. Trancone [23] used similar XRD and SEM experimental conditions in comparable studies to ensure result accuracy and comparability. (iii) An OPTIMA 8000DV inductively coupled plasma optical emission spectrometer from PerkinElmer was used to determine aluminum content in the acid leachate.

## 3. Results and Discussion

### 3.1. Effect of Calcination Temperature

**Figure 1:**
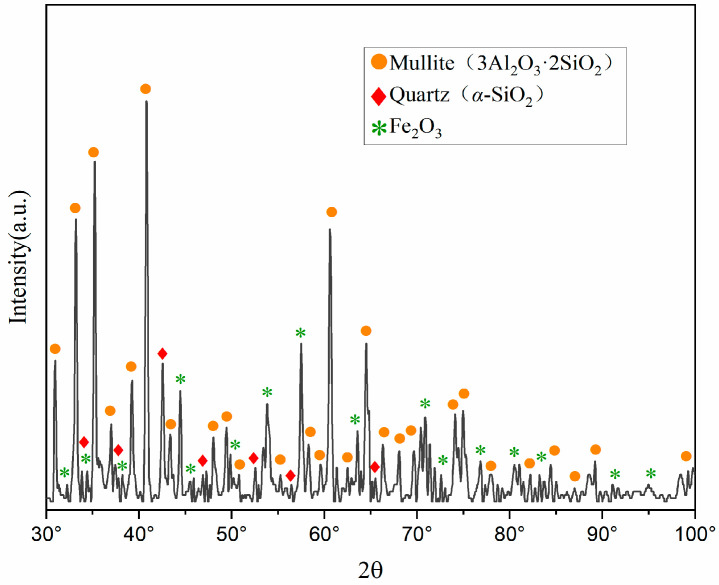
XRD pattern of pristine fly ash. (A quantity of FA was calcined with Na_2_CO_3_, and the resulting phases were compared against the JCPDS card database).

X-ray diffraction (XRD) characterization analysis was conducted on the pristine fly ash, with the mineral composition analysis presented in Figure 1. The mineral constituents of the fly ash are predominantly mullite (3Al_2_O_3_·2SiO_2_), quartz (*α*-SiO_2_), hematite (Fe_2_O_3_), and aluminosilicate glass phases. Notably, aluminum oxide is primarily present in the form of mullite (3Al_2_O_3_ 2SiO_2_) and aluminosilicate glass phases (M_2_(SiO_3_)_n_).

**Figure 2:**
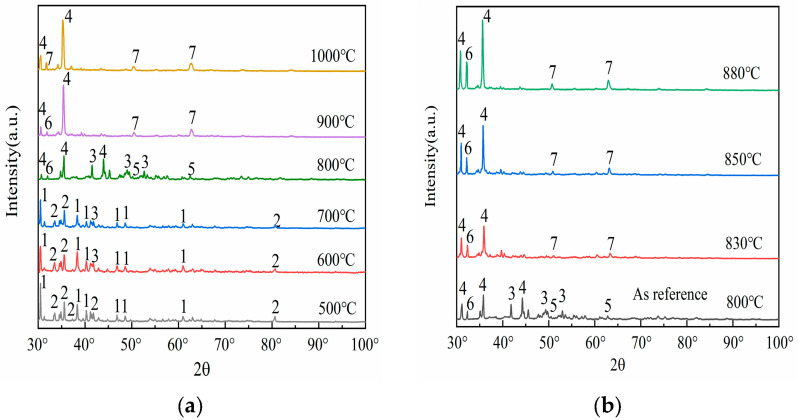
Under different temperatures, XRD patterns of the melt from fly ash and sodium carbonate mixtures. 1—Na_2_CO_3_, 2—Mullite (3Al_2_O_3_·2SiO_2_), 3—NaAlSiO_4_ (orthorhombic), 4—NaAlO_2_ 5—Na_2_Si_2_O_5_, 6—NaAlSiO_4_(hexagonal), 7—Na_2_SiO_3_.

To elucidate the phase transformation mechanism of mullite under the influence of sodium carbonate, this study employed X-ray diffraction (XRD) technology to identify the phases of the fused samples of fly ash mixed with sodium carbonate under various reaction temperature conditions. Figure 2 illustrates the phase changes obtained from the analysis.

The evolution of XRD diffraction peak intensities constitutes a composite manifestation of crystallographic integrity, phase composition, lattice defects, and chemical modifications. This intensity modulation is directly governed by structural reorganization within crystalline domains and/or surface functionalization processes. Three primary mechanisms govern this correlation: (i) crystallinity variation, (ii) phase transformations, and (iii) emergence of neoformed phases. As evidenced by the phase evolution diagram in Figure 2, a characteristic amorphous-to-crystalline transition occurs, after high-temperature roasting of fly ash; mullite (2, 3Al_2_O_3_·2SiO_2_, orthorhombic) precipitates from the glassy phase, with its crystal plane diffraction peak intensity increasing with higher crystallinity.

Upon comparing the results of Figure 1 with Figure 2, it is evident that as the calcination temperature gradually increases, the XRD diffraction peak intensities of both mullite (3Al_2_O_3_·2SiO_2_) and sodium carbonate (1, Na_2_CO_3_, orthorhombic) exhibit a declining trend. This phenomenon manifests directly in XRD diffraction patterns, where crystalline phase diffraction arises from the long-range ordering of lattice periodicity. In contrast, amorphization disrupts this structural coherence, resulting in progressive attenuation of long-range order parameters (LRO). Consequently, the corresponding diffraction peak intensity decreases significantly or disappears. Specifically, the diffraction peaks of sodium carbonate (Na_2_CO_3_) almost completely vanish above 700 °C. This phenomenon emerges in the 700–800 °C range, contrasting with the intrinsic decomposition threshold of pure sodium carbonate (>850 °C). The depressed decomposition temperature originates from four synergistic mechanisms: (i) Reactive interfacial coupling between Na_2_CO_3_ and aluminosilicate oxides (e.g., SiO_2_/Al_2_O_3_ in fly ash) generates intermediate sodium aluminosilicate (3, NaAlSiO_4_, orthorhombic), as evidenced by XRD peak evolution at 2θ = 42.6°; (ii) Eutectic melting induced by low-melting-point impurities (Fe_2_O_3_, CaO) creates localized liquid phases, accelerating intermediate formation via enhanced ionic mobility; (iii) Kinetic competition shows that sodium carbonate is not fully decomposed, but released CO_2_ and Na^+^ modify the silicoaluminate network, forming reactive new phases; (iv) Thermodynamically, intermediate phase formation (ΔG < 0) is spontaneous but must overcome the sodium carbonate decomposition energy barrier. These characteristics are used in low-temperature activation processes for aluminum recovery. Precise temperature control (700 °C) boosts acid leaching efficiency and avoids excessive high-temperature consumption. This demonstrates the environment-dependent thermal stability of Na_2_CO_3_ in complex systems, where actual decomposition pathways are governed by the chemo-geometric synergy between reactant activity and co-existing phases, while those of mullite (3Al_2_O_3_·2SiO_2_) become nearly invisible above 900 °C. This phenomenon is synergistically driven by three mechanistic dimensions: (i) Pyrolysis–chemical erosion coupling. Residual sodium carbonate or impurities (e.g., Na^+^) form low–melting liquid phases. These disrupt the aluminum–oxygen octahedra and silicon–oxygen tetrahedra structures, causing decomposition into corundum (α–Al_2_O_3_) and quartz (SiO_2_). (ii) Thermodynamically, corundum (α–Al_2_O_3_) and quartz have lower Gibbs free energy than mullite, making them more stable at high temperatures; (iii) In terms of kinetics, ionic diffusion in the liquid phase controls the decomposition rate. The disappearance of mullite reveals its relative thermal stability. Despite a melting point of 1890 °C, its structure is vulnerable to low–temperature attack in alkaline environments or when liquid phases are present. Industrially, temperature (e.g., below 900 °C) must be strictly controlled, or inhibitors added (e.g., pre–desilication to reduce SiO_2_ content) to prevent decomposition that increases aluminum recovery difficulty. Also, the metastable nature of mullite indicates that its behavior in high–temperature chemical processing requires considering both thermodynamic stability and kinetic conditions.

Observations from Figure 2a reveal that at a temperature of 500 °C, the XRD spectrum primarily displays the diffraction peaks of sodium carbonate (1, Na_2_CO_3_, orthorhombic) and mullite (2, 3Al_2_O_3_·2SiO_2_, orthorhombic), indicating that no significant chemical reaction has occurred between the two at this temperature. Upon raising the temperature to 600 °C, new diffraction peaks begin to emerge, with the formation of a peak for sodium aluminosilicate (3, NaAlSiO_4_, orthorhombic) at 42.6°, representing the initial product of the reaction between sodium carbonate (Na_2_CO_3_) and mullite (3Al_2_O_3_·2SiO_2_). Sodium aluminosilicate (the intermediate phase mentioned earlier) has a looser structure than the original silicoaluminate oxides (e.g., mullite or glassy phase). Its aluminum bonding state is more susceptible to disruption, significantly enhancing aluminum’s chemical reactivity and promoting aluminum leaching efficiency in subsequent acid leaching processes. As the temperature reaches between 600 °C and 700 °C, the diffraction peak of sodium aluminosilicate (NaAlSiO_4_) gradually intensifies, suggesting that increased temperature favors the phase transformation reaction of mullite (3Al_2_O_3_·2SiO_2_) under the influence of sodium carbonate (Na_2_CO_3_), that is, mullite becomes more susceptible to erosion by sodium carbonate and transforms into sodium aluminosilicate (NaAlSiO_4_). When the temperature rises to 800 °C, multiple new diffraction peaks appear in the XRD spectrum, indicating the formation of additional phases, including the peaks for sodium metaaluminate (4, NaAlO_2_, orthorhombic) at 32.7°, disodium disilicate (5, Na_2_Si_2_O_5_, orthorhombic) at 51.2°, and nepheline (6, NaAlSiO_4_, hexagonal) at 33.1°. As the temperature continues to increase to 900 °C, the diffraction peaks for sodium metasilicate (7, Na_2_SiO_3_, hexagonal) at 51.2° and 63.3° begin to manifest. Further raising the temperature to 1000 °C results in a stable diffraction pattern, indicating that the reaction between mullite (3Al_2_O_3_·2SiO_2_) and sodium carbonate (Na_2_CO_3_) has reached a relatively stable state. Experimental studies by A. Molina [24] revealed that when the reaction temperature exceeds 900 °C, the activation efficiency of coal fly ash exhibits a paradoxical decrease rather than enhancement, which can be attributed to high-temperature-induced lattice reconstruction of fly ash components—a process predominantly occurring within the 900–1300 °C temperature range. This phenomenon can be attributed to the lattice reconstruction of fly ash components induced by high temperatures. Therefore, it is clear that during the activation process of fly ash, the increase in temperature does not follow a linear enhancement pattern, meaning that higher temperatures do not necessarily yield better activation effects. After the above structural discussion and considering the reduction in energy consumption, combined with spectral information, it is evident that the activation effect of mullite within the calcination temperature range of 800 to 900 °C is optimal. Hence, further detailed research on phase transformation within this temperature range is warranted.

Following the aforementioned discussion, a detailed study of the phase changes in the fused materials at calcination temperatures of 830 °C, 850 °C, and 880 °C was conducted, with the XRD patterns presented in Figure 2b. Referring to the XRD pattern of the fused material at a calcination temperature of 800 °C, upon further elevation of the temperature, it becomes evident that the peak intensities of sodium metaaluminate (NaAlO_2_) and nepheline (NaAlSiO_4_) also continuously rise, and the diffraction peaks of sodium metasilicate (Na_2_SiO_3_) become increasingly distinct. This indicates that the activation reaction between sodium carbonate and fly ash is still underway within the 800–900 °C range, eventually reaching a relatively stable state. The specific manifestations are as follows: when the calcination temperature reaches 830 °C, the diffraction peaks of sodium metasilicate (Na_2_SiO_3_) begin to emerge, particularly evident at 63.8°; upon further increasing the temperature, it is found that at a calcination temperature of 850 °C, the peak intensity of nepheline (NaAlSiO_4_) continues to increase, and the diffraction peaks of sodium metasilicate (Na_2_SiO_3_) also gradually become apparent; as the temperature rises to 880 °C, the diffraction peak of nepheline (NaAlSiO_4_) essentially reaches its peak value, suggesting that this temperature is sufficient for sodium carbonate (Na_2_CO_3_) to fully activate the fly ash. Upon synthesis of the experimental findings and ensuing deliberations, it has been concluded that a calcination temperature of 880 °C is optimal for conducting XRD analysis to assess the impact of varying material ratios.

The investigation into high-temperature interactions between sodium carbonate (Na_2_CO_3_) and mullite (3Al_2_O_3_·2SiO_2_) has elucidated their synergistic thermokinetic mechanisms, providing critical optimization pathways for industrial and technological applications. This fundamental understanding enables precise control of phase evolution processes while balancing energy efficiency and material performance. In aluminum recovery, precise temperature control (700 °C for reactive intermediate phase formation, avoiding mullite decomposition at 900 °C) boosts efficiency and cuts energy use. In refractory and ceramic industries, mullite stability is maintained by avoiding alkaline conditions, or new functional materials are developed using intermediate phases. In metallurgy, sodium carbonate’s eutectic effect lowers smelting temperatures, and by–product silicate slags are resourcefully used (e.g., in construction materials), promoting circular economy models. These findings, by balancing thermodynamic drives and kinetic constraints, help industries enhance energy efficiency, reduce costs, and achieve environmental goals, advancing resource valorization and sustainable development.

### 3.2. Effect of Material Ratio

**Figure 3:**
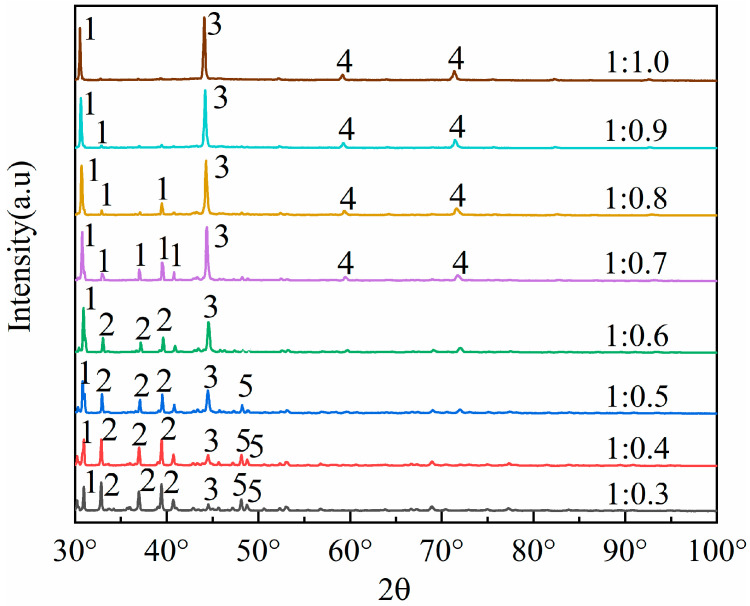
Different mass ratios of fly ash and sodium carbonate melt XRD patterns. 1—Nepheline (NaAlSiO_4_), 2—Mullite (3Al_2_O_3_·2SiO_2_), 3—NaAlO_2_, 4—NaAlSiO_4_, 5—SiO_2_.

To cleave the silicoaluminate bonds within mullite (3Al_2_O_3_·2SiO_2_), sodium carbonate is selected as an activating agent to react with aluminum oxide and silicon dioxide, thereby generating nepheline (NaAlSiO_4_), which is more soluble in acid. This process aims to enhance aluminum extraction through the activation roasting with sodium carbonate. Studies show that the fly ash–sodium carbonate ratio significantly impacts activation efficiency. At low sodium carbonate ratios (1:0.3 to 1:0.5), insufficient carbonate reacts with Al_2_O_3_ and SiO_2_ in fly ash. This prevents the full conversion of the aluminosilicate glass phase to the reactive intermediate phase (NaAlSiO_4_), leaving mullite and resulting in an aluminum–leaching rate of less than 65%. At high ratios (1:0.8 to 1:1.0), excess carbonate creates a high–alkalinity liquid phase, causing excessive sintering of fly ash particles. The resulting dense particles hinder acid penetration and produce by–products like sodium silicate, which are detrimental to leaching. Consequently, thise experiment selected fly ash–sodium carbonate ratios of 1:0.3, 1:0.4, 1:0.5, 1:0.6, 1:0.7, 1:0.8, 1:0.9, and 1:1.0, which were used for the roasting activation. Subsequently, the fused materials with varying ratios of fly ash to sodium carbonate were characterized using XRD analysis, with the results presented in Figure 3.

By comparing the XRD patterns of pristine fly ash (Figure 1) with those of the fused materials after calcination activation (Figure 3), it is evident that the degree of reaction between mullite (3Al_2_O_3_·2SiO_2_) in the fly ash and the activating agent, sodium carbonate, varies as the amount of sodium carbonate (Na_2_CO_3_) increases, leading to a gradual decrease in mullite (3Al_2_O_3_·2SiO_2_) until it disappears. When the material ratio of FA to Na_2_CO_3_ is between 1:0.3 and 1:0.5, mullite mineral (3Al_2_O_3_·2SiO_2_) partially transforms into nepheline, yet a portion remains as the mullite mineral (3Al_2_O_3_·2SiO_2_) phase. In this stage, the insufficient amount of sodium carbonate restricts the reaction chemically, resulting in a low aluminum leaching rate of only 32% to 65%. The calcination activation product is reddish-brown, loosely textured, and easily removable, with no sintering occurring. At a material ratio of 1:0.6, mullite (3Al_2_O_3_·2SiO_2_) is largely converted into nepheline (NaAlSiO_4_, hexagonal), as indicated by the increased presence of nepheline in the XRD patterns. When the ratio is 1:0.7, mullite (3Al_2_O_3_·2SiO_2_) is entirely transformed into nepheline (NaAlSiO_4_, hexagonal), suggesting that the amount of sodium carbonate (Na_2_CO_3_) is sufficient to fully activate the mullite (3Al_2_O_3_·2SiO_2_) in the fly ash. At this ratio (Na_2_CO_3_/FA ≈ 1:0.6 to 1:0.7), sodium reacts fully with silicoaluminate components, achieving an aluminum leaching rate of 66% to 85%. The calcination activation product is grayish-yellow, soft in texture, and easily removable, with no sintering. At a ratio of 1:0.8, mullite (3Al_2_O_3_·2SiO_2_) disappears entirely, giving rise to nepheline (NaAlSiO_4_, hexagonal) and sodium aluminosilicate (NaAlSiO_4_), with the calcination activation product being predominantly yellow with some green areas, denser in texture, more difficult to remove, and exhibiting sintering. When the material ratio reaches 1:0.9 and 1:1.0, the XRD pattern peaks are essentially the same as at the 1:0.8 ratio, indicating that the reaction has reached a stable state. The calcination activation product is yellow-green, compact in texture, difficult to remove, and sintered. Excess sodium carbonate creates a high-alkalinity liquid phase that encapsulates particles, triggers side reactions (e.g., sodium silicate formation), and increases acid leaching difficulty, decreasing the leaching rate to 70–75%.

**Figure 4:**
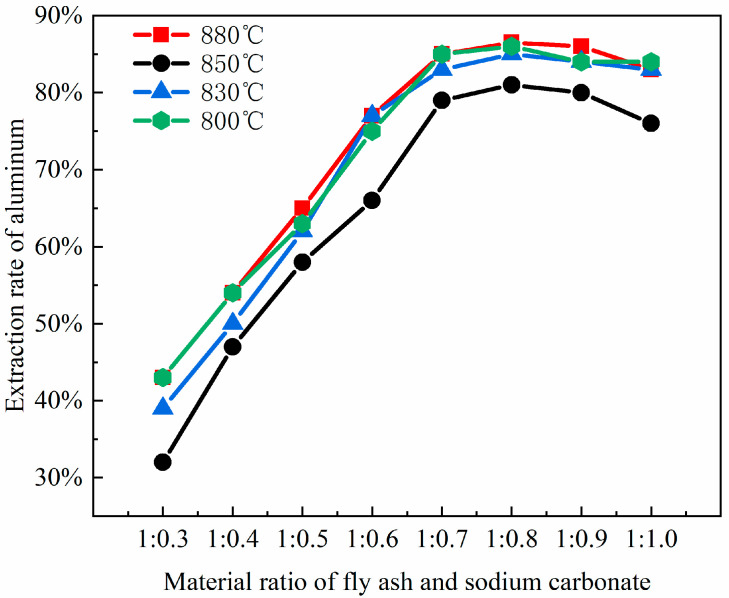
The impact of different material ratios on aluminum extraction rates.

Notably, sodium carbonate loss (e.g., high-temperature volatilization, reactions with Fe_2_O_3_/CaO impurities) further impacts efficiency. For instance, a 1% increase in CaO content raises sodium loss by about 3%, necessitating a 20–30% excess addition of sodium carbonate to compensate for increasing costs. Thus, an experimental fly ash–sodium carbonate ratio of 1:0.7 achieves the highest aluminum leaching rate (Figure 4). Higher ratios, though completing mullite conversion, reduce efficiency due to side reactions of by-products (e.g., sodium aluminosilicate does not react with acid; nepheline is acid-soluble), their accumulation, and sintering. To enhance cost-effectiveness, pre-desilication (reducing SiO₂ content) or mineralizers (e.g., CaF₂) can be introduced, lowering the calcination temperature from 750 °C to 650 °C in order to reduce sodium loss.

XRD analysis shows that the fly ash–sodium carbonate ratio affects the product phase composition. At a 1:1 ratio and 880 °C calcination, nepheline forms, boosting aluminum extraction. Excess or insufficient sodium carbonate generates by-products like sodium silicoaluminate or leaves unreacted minerals. Optimizing the material ratio to 1:1 and keeping the calcination temperature around 700 °C reduces by-products and environmental impact. Precise control of the material ratio and impurity management are key to balancing aluminum recovery and process costs. As the ratio of coal fly ash to sodium carbonate increases, the extraction yield of alumina exhibits an initial increase followed by a subsequent decrease. The leaching efficiency of alumina reaches its zenith when the material ratio of coal fly ash to sodium carbonate is 1:0.8, which is in substantial agreement with the phase transformation patterns of the coal fly ash depicted in Figure 3.

### 3.3. Effect of Roasting Time

**Figure 5:**
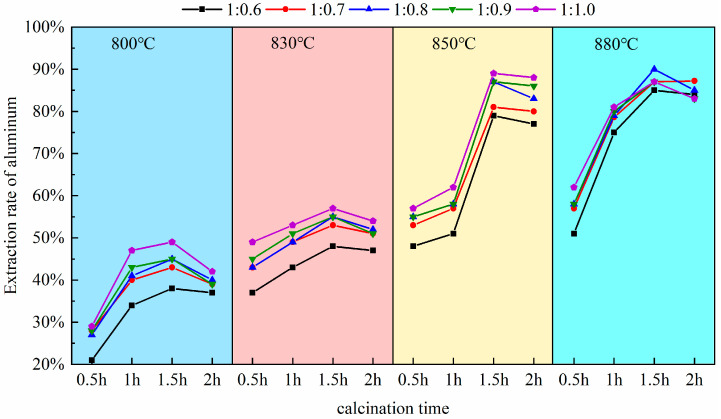
The impact of calcination time on aluminum extraction rate.

Pursuant to this experiment conducted at a calcination temperature of 880 °C, as established in Section 3.1, the calcination durations were selected to be 0.5 h, 1.0 h, 1.5 h, and 2.0 h. Upon completion of the reactions, the molten products were allowed to cool naturally and then retrieved. Subsequently, the extraction yield of alumina was measured and calculated following the acid leaching, stirring, and solid–liquid separation procedures outlined in the experimental methods of Section 2.2. To eliminate experimental anomalies and ensure result reliability, multiple experimental controls and repetitions, shown in Figure 5, were set up. These considered calcination temperature, time, and material ratio. Three parallel experiment groups were obtained. For example, at a material ratio of 1:0.8 and 830 °C calcination temperature, the extraction rate after 1.5 h (54%) was significantly higher than after 1 h (45%).

As depicted in Figure 5, the calcination duration exerts a significant influence on the activity of alumina within coal fly ash. At excessively high temperatures (e.g., 2 h), the aluminum extraction rate decreases. This is due to the sintering of particle surfaces from prolonged high-temperature exposure, causing densification. Also, excess sodium salts (Na^+^) react with free SiO_2_ to form by-products (sodium aluminosilicates), which do not react with acid. Overall, the key mechanism for the decrease in reactivity is a cascade effect of “kinetic hindrance (sintering) → by-product formation → thermodynamic reverse reaction”, resulting in a trend where the extraction yield of aluminum first increases and then decreases. Within the range of 0.5 h to 1.5 h of calcination, the gradient of the extraction rate curve is in the steeply ascending segment; there is a direct correlation between the extended duration and the increased reactivity of alumina, which corresponds to a notable improvement in the extraction yield of aluminum, a pattern especially pronounced between 0.5 h and 1.0 h. Within this interval, the curve shows high increments and a steep slope. The aluminum extraction rate increases rapidly from 57% to 79% at a calcination temperature of 880 °C and a material ratio of 1:0.7. This indicates that increasing the calcination time significantly promotes aluminum extraction, with a substantial increase in extraction rate. The data align with a first-order reaction kinetics model, suggesting that the reaction rate is dominated by interfacial chemical reactions. The solid-solid reaction between sodium carbonate and mullite rapidly forms nepheline. With the augmentation of calcination time up to 1.2 h, the curve maintains an upward trend (Plateau stage), yet the gradient is less steep than in the 0.5 h to 1.0 h range; this process conforms to the Ginstling–Brounshtein diffusion model, indicating that the reaction becomes limited by the diffusion rate of Al^3+^/Na^+^ through the product layer; Upon reaching a calcination time of 1.5 h, the extraction yield essentially attains its maximum value, observable in the figure by a markedly diminished increment in the curve and an approximate leveling of the slope. Based on the above discussion and considering extraction efficiency, energy consumption, and environmental impact, 1.5 h was chosen as the optimal calcination time. Experimental data shows that at 1 h, the aluminum leaching rate peaks at 83% (at 880 °C). At 1.5 h, it marginally increases to 89%. However, the latter ensures complete mullite–to–nepheline conversion (XRD residual peak intensity < 1%), preventing unreacted cores from interfering with subsequent acid leaching. Additionally, a longer calcination time reduces acid–leaching effluent and by–products (e.g., silica gel), enhancing net environmental benefits. Thus, 1.5 h achieves the best balance among extraction rate, cost, and sustainability.

**Figure 6:**
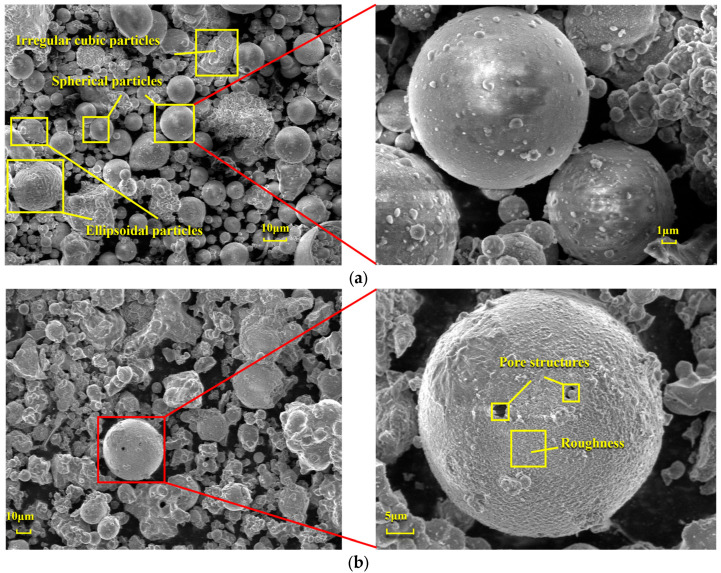
SEM Images of FA: (**a**) Before calcination activation and (**b**) After calcination activation.

**Figure 7:**
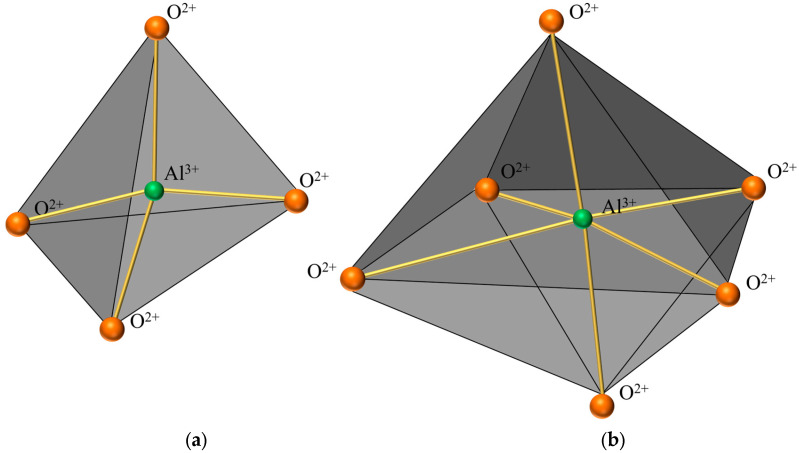
Crystal structure diagram. (**a**) Aluminum-oxygen tetrahedral crystal structure. (**b**) Aluminum-oxygen octahedral crystal structure.

**Figure 8:**
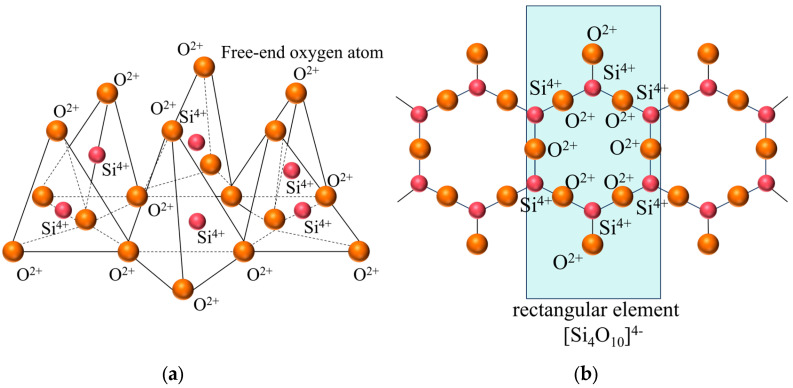
Layered silicon (aluminum) oxygen tetrahedron. (**a**) stereogram (**b**) Z-axis projection.

Figure 6 presents a comparison of scanning electron microscope (SEM) images of pristine fly ash and those under specific calcination activation conditions. As shown in Figure 6a, the microparticles of pristine fly ash predominantly exhibit spherical, elliptical, and irregular cubic shapes with surfaces that are approximately smooth. Figure 6b, in contrast, displays the SEM image obtained under experimental conditions of a calcination temperature of 880 °C, a material ratio of 1:0.7, and a calcination time of 1.5 h. Compared to Figure 6a, the calcined fly ash particles exhibit a marked increase in porosity, a more fluffy surface structure, and a significant transformation in crystal structure. These observations suggest that during the calcination activation process, the aluminate octahedral crystal structure in fly ash underwent the cleavage of Al-O bonds, subsequently transforming into an silicon (aluminum) oxygen tetrahedron crystal structure (as depicted in Figure 7). The structural transition from octahedral to tetrahedral coordination in aluminates induces the formation of six-membered rings (Figure 8), where interconnected AlO_4_ and SiO_4_ tetrahedra construct [Si_4_O_10_]^4−^ rectangular units, significantly enhancing acid susceptibility. In the octahedral configurations, aluminum ions are surrounded by six oxygen atoms, forming a stable coordination environment. This structure is relatively dense and resistant to acid attack. In the tetrahedral structure, aluminum ions are surrounded by four oxygen atoms, reducing the coordination number and stability. This transformation enhances the chemical reactivity of aluminum salts in acidic environments, making them more susceptible to acid attack. These ring structures serve as basic units for constructing aluminosilicate materials, such as nepheline (acid-soluble).

### 3.4. Effect of Hydrochloric Acid Concentration

The concentration of hydrochloric acid is crucial in the acid–leaching process, which directly impacts the leaching kinetics and reaction pathways of aluminum. In the low-concentration range (1–3 mol/L), insufficient H^+^ concentration leads to inefficient disruption of the Al-O bonds in nepheline (NaAlSiO_4_). The reaction is controlled by surface chemical reactions, with the reaction rate linearly related to the acid concentration. At medium–high concentrations (4–6 mol/L), the synergistic effects of H^+^ and Cl^−^ become significant. H^+^ attacks the nepheline structure to release Al^3+^, while Cl^−^ promotes aluminum ion dissolution through complexation (forming AlCl_4_^−^). Leaching efficiency can reach 80–90%, and the reaction shifts to diffusion control, with the rate limited by ion migration. When the concentration exceeds 6 mol/L (7–8 mol/L), high acidity triggers a surge in side reactions. These include the competitive dissolution of impurity ions like Fe^3+^ and Ca^2+^ and the rapid formation of silica gel (SiO_2_·nH_2_O). The silica gel encapsulates unreacted particles, creating a diffusion barrier and reducing the aluminum leaching rate to below 85%, and higher concentrations of hydrochloric acid not only have strong volatility but may also lead to overly vigorous reactions, resulting in significant losses of volatile chemicals. This not only increases costs but also has adverse effects on the leaching operation and the environment. Particularly during acid leaching at high concentrations of hydrochloric acid, a substantial amount of aluminum silicate sol is produced, which makes the solid-liquid separation process extremely difficult. However, should the hydrochloric acid’s concentration fall below the optimal level, it can result in insufficient acid leaching reactions, thereby affecting the extraction rate of aluminum.

**Figure 9:**
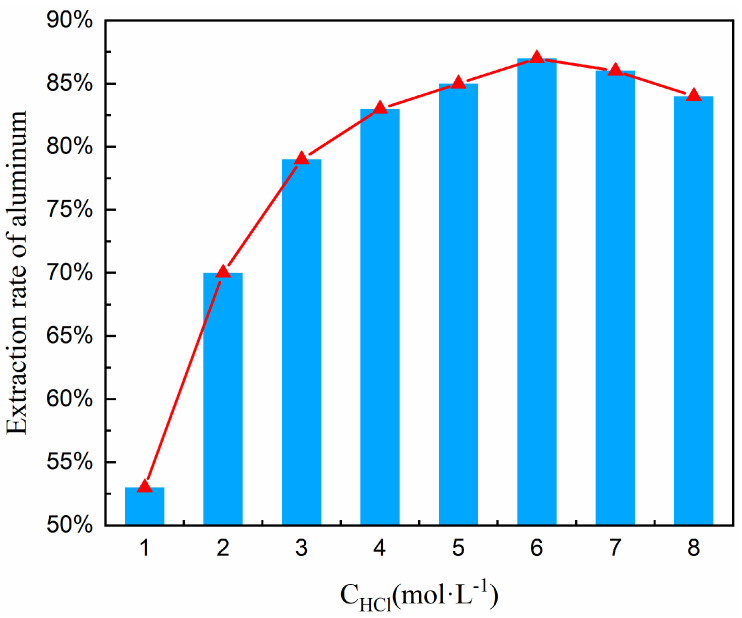
Effect of hydrochloric acid concentration on aluminum extraction rate.

To determine the optimal hydrochloric acid concentration, a systematic concentration variation experiment was conducted within the range of 1–8 mol/L under precisely controlled thermal conditions (100 °C); the lower limit of 1 mol/L ensures the minimum effective H^+^ concentration to initiate the acidolysis of aluminosilicates, avoiding ineffective leaching due to insufficient acid. The upper limit of 8 mol/L balances diminishing returns and operational risks. Beyond 8 mol/L, aluminum–leaching efficiency plateaus occur, while acid consumption and waste liquid treatment costs (e.g., neutralization and recovery) rise. High-concentration acid also significantly accelerates the corrosion rate of reactor materials like titanium alloys. Moreover, this range encompasses the entire kinetics of aluminum leaching: low concentrations (1–3 mol/L) correspond to chemically-controlled reactions, medium concentrations (4–6 mol/L) to diffusion-controlled reactions, and high concentrations (7–8 mol/L) reveal extreme conditions dominated by side reactions (e.g., silica gel formation), providing comprehensive data for process optimization. Other experimental conditions included a material ratio of FA to Na_2_CO_3_ of 1:0.7, a calcination temperature of 880 °C and a calcination time of 1.5 h (obtained from Section 3.3); the hydrochloric acid solution was maintained at 100 °C, a solid-liquid ratio at 1:5, and a leaching time at 1 h. Under these established conditions, a series of experiments were executed to explore the influence of varying hydrochloric acid concentrations on the aluminum extraction rate. The findings of these experiments are delineated in Figure 9.

Based on the trend line in Figure 9, it has been noted that the rate of aluminum extraction exhibits an upward trend followed by a decline as the concentration of hydrochloric acid escalates. This reveals the nonlinear relationship between acid concentration and aluminum leaching rate, as well as the critical point for by-product formation: (i) The leaching rate peaks at 83–86% in the 4–6 mol/L range, indicating optimal diffusion-controlled conditions where the reaction rate is limited by the diffusion of Al^3+^ from the particle surface to the liquid phase. (ii) After the concentration exceeds 6 mol/L, the leaching rate decreases (86% → 82%), signaling by-product formation (silica gel). The accumulation of silica gel is the main cause of the efficiency drop. Therefore, 6 mol/L hydrochloric acid was chosen for subsequent experiments.

### 3.5. Effect of Solid–Liquid Ratio

**Figure 10:**
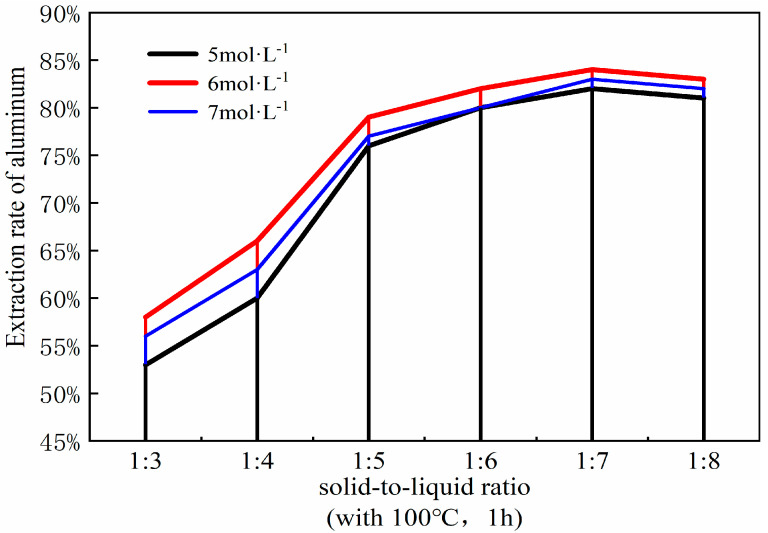
Effect of different solid-liquid ratios on aluminum extraction rate. (Acid leaching temperature 100 °C; acid leaching time 1 h).

The solid-to-liquid ratio is a pivotal parameter in the acid process of coal fly ash, influencing both the efficiency of mixing and energy dissipation, as well as the efficacy of aluminum extraction. Optimizing the solid-to-liquid ratio can refine the acid leaching procedure and augment the yield of aluminum extraction. To ascertain the optimal solid-liquid ratio for the acid leaching process, it was manipulated within the range of 1:3 to 1:8. The ancillary experimental parameters were set as follows: a coal fly ash to sodium carbonate ratio of 1:0.7, a calcination temperature of 880 °C, a calcination duration of 1.5 h, followed by leaching with 6 mol/L hydrochloric acid at 100 °C for a duration of 1 h. The resultant curve illustrating the impact of varying solid-liquid ratios on the aluminum extraction rate is presented in Figure 10.

At a solid-liquid ratio of 1:3, the aluminum content within the fly ash fails to react completely, leading to a suboptimal yield of aluminum oxide. However, it is noteworthy that elevating the solid-liquid ratio to 1:4 markedly enhances the extraction efficiency of aluminum oxide, surpassing the results obtained at the 1:3 ratio. This is attributed to the decrease in the solid-liquid ratio, effectively increasing the amount of hydrochloric acid, which, according to the principles of chemical equilibrium, favors the progress of the reaction and increases the reaction rate. Hence, a moderate augmentation of the solid-liquid ratio is instrumental in bolstering the extraction rate of aluminum. When the solid-liquid ratio reaches 1:5, the extraction rate of the aluminum shows a particularly noticeable increase compared to the 1:4 ratio. However, beyond a solid-liquid ratio of 1:5, the increase in the extraction rate of aluminum oxide markedly decreases, essentially reaching an equilibrium state, with the rate of aluminum extraction gradually slowing down and stabilizing. At a solid-liquid ratio of 1:6, the data plotted on the curve suggest that the rate of aluminum extraction has essentially plateaued, indicating the point of maximum efficiency. Furthermore, the variation curve shows that when the solid-liquid ratio increases to 1:7, the extraction rate of aluminum begins to slowly decline, a trend that continues up to 1:8. This is because an excessively high solid-liquid ratio not only causes other components in the fly ash to react with the excess hydrochloric acid, introducing impurities, but also leads to the waste of hydrochloric acid solution. Therefore, considering all factors, a solid-liquid ratio of 1:6 is selected for the experiment [25].

### 3.6. Effect of Leaching Time

**Figure 11:**
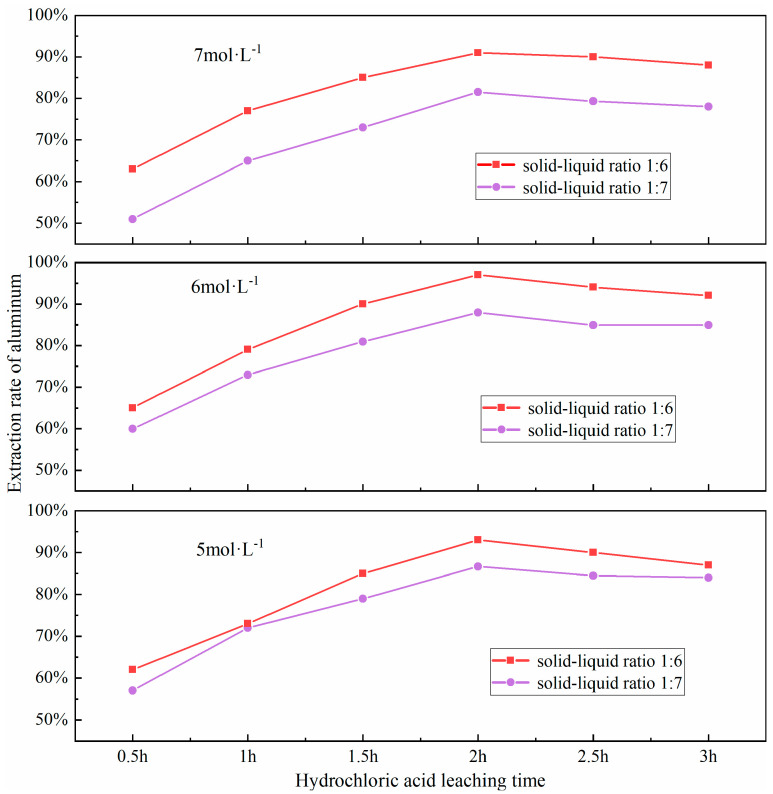
Effect of different hydrochloric acid leaching time on aluminum extraction rate.

The efficacy of the acid-leaching process is predominantly shaped by the extent of the leaching duration. If the leaching time is too short, the acid leaching reaction will not be sufficiently carried out; conversely, if the leaching time is too long, it may lead to a decrease in leaching efficiency. The underlying mechanism involves a dual process: (i) kinetics of leaching regime transition from chemical reaction control to diffusion control, and (ii) cumulative side reactions. This transition can be delineated into two distinct phases: Initial phase: Rapid chemical dissolution dominates, where active aluminum-bearing phases (e.g., NaAlSiO_4_, γ-Al_2_O_3_) react exergonically with HCl, resulting in time-dependent leaching efficiency enhancement (dC/dt > 0.85 min^−1^). Terminal phase: Progressive accumulation of reaction byproducts (e.g., residual silicates, neoformed sulfates) generates a diffusion-limiting passivation layer, transitioning the rate-limiting step to solid-state diffusion. Concurrently, prolonged leaching induces competitive dissolution of ancillary minerals (e.g., hematite Fe_2_O_3_, lime CaO), releasing Fe^3+^ and Ca^2+^ ions that either (a) compete with Al^3+^ for complexation sites or (b) precipitate as colloidal hydroxides (e.g., Fe(OH)_3_), thereby reducing aluminum recovery efficiency by 12–18%. Furthermore, selective dissolution occurs during acid treatment, where activated aluminum phases (e.g., nepheline NaAlSiO_4_) are preferentially leached, leaving refractory phases (e.g., mullite 3Al_2_O_3_·2SiO_2_, quartz SiO_2_) with enhanced structural stability. Based on this mechanistic understanding, a time-dependent leaching profile was established through systematic experiments at 0.5 h intervals across the 0.5–3.0 h range. These trials were conducted under a set of controlled conditions: a precise material ratio of fly ash to sodium carbonate at 1:0.7, calcination at a consistent temperature of 880 °C for a duration of 1.5 h, a hydrochloric acid solution was maintained at 100 °C and had a concentration of 6 mol/L; a solid-liquid ratio optimized to 1:6. The impact of different leaching times on the aluminum extraction rate was investigated, with the results presented in Figure 11.

As shown in Figure 11, the extraction rate of aluminum increases with the extension of the acid leaching time, reaching a peak when the leaching time is 2.0 h. However, a continued increase in leaching time results in a decline in the extraction rate, which may be attributed to the re-precipitation of dissolved aluminum due to excessive leaching times, thereby reducing the leaching efficiency. Upon examining the figure, it becomes apparent that a substantial surge in the extraction rate of aluminum is observed. when the leaching time is between 0.5 to 1.0 h and 1.0 to 1.5 h. This indicates that an appropriate leaching time can ensure a complete reaction of aluminum in FA, thereby enhancing the leaching rate of aluminum.

**Figure 12:**
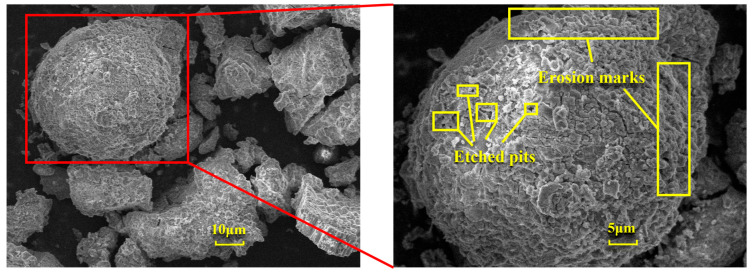
SEM images of FA before and after acid leaching.

Figure 12 illustrates the changes in the surface microstructure of FA after acid leaching, as observed through scanning electron microscopy (SEM). Compared with the SEM image of pristine FA in Figure 6a, it is evident that after hydrochloric acid treatment, the spherical particles exhibit distinct signs of erosion, with etched pits forming and the surface becoming rough. These alterations indicate that the acid leaching process disrupts the mullite structure within the fly ash, thereby releasing the aluminum elements contained within. The reaction mechanism is as follows: Calcination activation of fly ash under alkaline conditions provided by sodium carbonate leads to the cleavage of Al-O bonds in the aluminate octahedral crystal structure, which are then rearranged to form an aluminate tetrahedral crystal structure. Under the above alkaline pretreatment conditions, the reaction forms soluble aluminate phases. This creates more active sites on the mineral surface, providing additional reaction points for subsequent acid leaching and enhancing the etching efficiency. The pretreatment under alkaline conditions can partially dissolve inert phases on the mineral surface, such as mullite. This increases the mineral’s porosity and specific surface area, allowing acid to infiltrate deeper into the mineral and boosting the etching depth. Under the influence of hydrochloric acid (H^+^), it chemically reacts with the aluminosilicate minerals in fly ash. Specifically, hydrochloric acid reacts with the alumina and silicate components in these minerals to form soluble aluminum and silicate salts. This disrupts the original structure of the fly ash, causing its dense glassy surface to be etched and leading to the breakage of Al-O bonds and the release of aluminum ions. As the Al-O bonds cleave, etching pits gradually form on the fly ash surface. These pits increase the specific surface area of the solid particles and expose more active sites, further promoting the reaction between the acid and the minerals and accelerating the dissolution of fly ash and the extraction of aluminum.

In summary, the microstructural changes in fly ash after treatment affect its overall properties and potential applications as follows: (i) The formation of etching pits and an increased specific surface area enhance the chemical reactivity of fly ash, making it more responsive to subsequent chemical treatments and improving the extraction rate of aluminum and other valuable elements. (ii) Microstructural alterations can influence the physical properties of fly ash, such as particle morphology, size, and porosity, which may affect its performance as a filler, adsorbent, or other functional materials. (iii) Microstructural changes can also impact the environmental effects of fly ash. For example, an increased specific surface area and porosity may enhance its adsorption capacity for pollutants, offering potential applications in environmental remediation.

### 3.7. Reproducibility and Statistical Validation of Alumina Extraction from Fly Ash Using Alkali

To accurately assess the interactions between factors, an orthogonal experimental design is used: (i) The orthogonal table is designed based on six factors affecting aluminum extraction from fly ash: calcination temperature, material ratio, calcination time, hydrochloric acid concentration, solid-liquid ratio, and acid–leaching time. These factors have significant interactions. (ii) The L18 (3^6^) orthogonal array is chosen for quantitative analysis. Range analysis evaluates interaction effects, and significant interactions are further analyzed. (iii) Model validation shows R^2^ = 0.959, indicating that the six main factors and their potential interactions explain 95.9% of the variation. Columns are assigned to interaction terms in the orthogonal table, and their R and F values are calculated. (iv) Statistical tests verify the significance of Kavg differences using ANOVA and post-hoc tests to confirm significant differences at different levels.

The principle of factor and level selection: (i) Factor selection, six parameters significantly affecting aluminum extraction rate were chosen: calcination temperature (A: 830 °C, 850 °C, 880 °C), material ratio (B: 1:0.7, 1:0.8, 1:0.9), calcination time (C: 1 h, 1.5 h, 2 h), hydrochloric acid concentration (D: 5 mol/L, 6 mol/L, 7 mol/L), solid-liquid ratio (E: 1:6, 1:7, 1:8), acid-leaching temperature (E: Maintain at 100 °C) and acid-leaching time (G: 1.5 h, 2 h, 2.5 h), with effective ranges determined by preliminary experiments; (ii) Level setting, levels cover the operable interval (three levels); for example, the calcination time spans 0.5 h to ensure the experimental design can capture parameter effects; (iii) Orthogonal table matching; the appropriate orthogonal array (L_18_3^6^) was chosen as the control parameter design for quantitative analysis, and repeat experiments were conducted. Table 2 shows the controlled factors and levels for the (L_18_3^6^) orthogonal array, with the extraction rate as the orthogonal experiment response.

Table 3 uses the Tukey HSD test to compare differences between levels. For instance, the extraction rate at 880 °C calcination temperature is significantly higher than at 830 °C (97% in experiment 13 vs. 83.16% in experiment 1). As indicated by the results in Table 3, an alumina extraction efficiency of up to 97% can be achieved when the fly ash-to-sodium carbonate mass ratio is 1:0.7, after calcination at 880 °C for 1.5 h, this pretreatment was followed by leaching with 6 mol/L hydrochloric acid at 100 °C under controlled solid-liquid phase conditions (1:6 ratio) for 2 h. Under the aforementioned experimental conditions, the extraction efficiency of alumina can reach 97%.

Analysis of range (range analysis) of orthogonal experimental results is an important method for evaluating the experimental index values at each level of the factors, examining the interactions between factors, determining the significance of different factors on the experimental outcomes, and ultimately identifying the optimal combination of factor levels that maximizes the experimental index. Table 4 presents the range analysis table, which includes the sum of the experimental index values at the ith level of a factor (K_i_), the number of replicates per level (r), the number of levels for a factor (m), the average experimental index at the same level (K_avg_), and the optimal level (range) R. R denotes the range value of a factor, which is calculated as the difference between the maximum and minimum values of Kavg for that factor. The range values can be used to compare the relative importance of different factors. It reflects the degree of impact on experimental results when factor levels change. (i) Factors can be ranked by R-value size; those with larger R-values significantly affect results and are main factors, while those with smaller R-values have less impact and are secondary factors. (ii) Numerical-difference analysis quantitatively determines factor importance by reflecting the relative intensity of different factors’ impacts on results. For example, the sum of the experimental index values (K_1_) and the corresponding average experimental index (K_avg_) at the first level of factor C are calculated in Equations (2) and (3), where L_i_ represents the response in the ith row of the orthogonal experimental table.

(2) $$K_{1}=L_{1}+L_{3}+L_{7}+L_{11}+L_{15}+L_{17}$$

(3) $$K_{1-\mathrm{avg}}=\frac{L_{1}+L_{3}+L_{7}+L_{11}+L_{15}+L_{17}}{r}$$

For managing and assessing intra–replicate variability, take factor C as an example: (i) Calculate variability metrics using Formulas (2) and (3) to quantify intra-replicate variability, which usually involves calculating statistical measures like standard deviation or variance to numerically represent data dispersion; (ii) Manage variability by multiple replicates (e.g., repeating experiment 13 three times) to reduce random errors and enhance data stability. Repeating experiments improves estimation and control of variability, making results more representative and reliable; (iii) Discuss the impact on the optimal combination. High variability may mask true effects, making it difficult to accurately identify interactions and main effects between factors in experiments. However, orthogonal experiments can effectively reduce the impact of variability through repeated design (e.g., six repeats per level) and mean calculation, ensuring the reliability of the optimal parameter combination. For instance, in Table 3, experiment 13 achieves an extraction rate of 97% under the following conditions: a material ratio of 1:0.7, a calcination temperature of 880 °C, a calcination time of 1 h, a solid–liquid ratio of 1:7, a hydrochloric acid concentration of 7 mol/L, an acid-leaching temperature of 100 °C, and an acid–leaching time of 2 h. This indicates that proper experimental design and data processing can overcome the interference of intra-replicate variability in identifying the optimal combination and finding the best experimental conditions.

**Figure 13:**
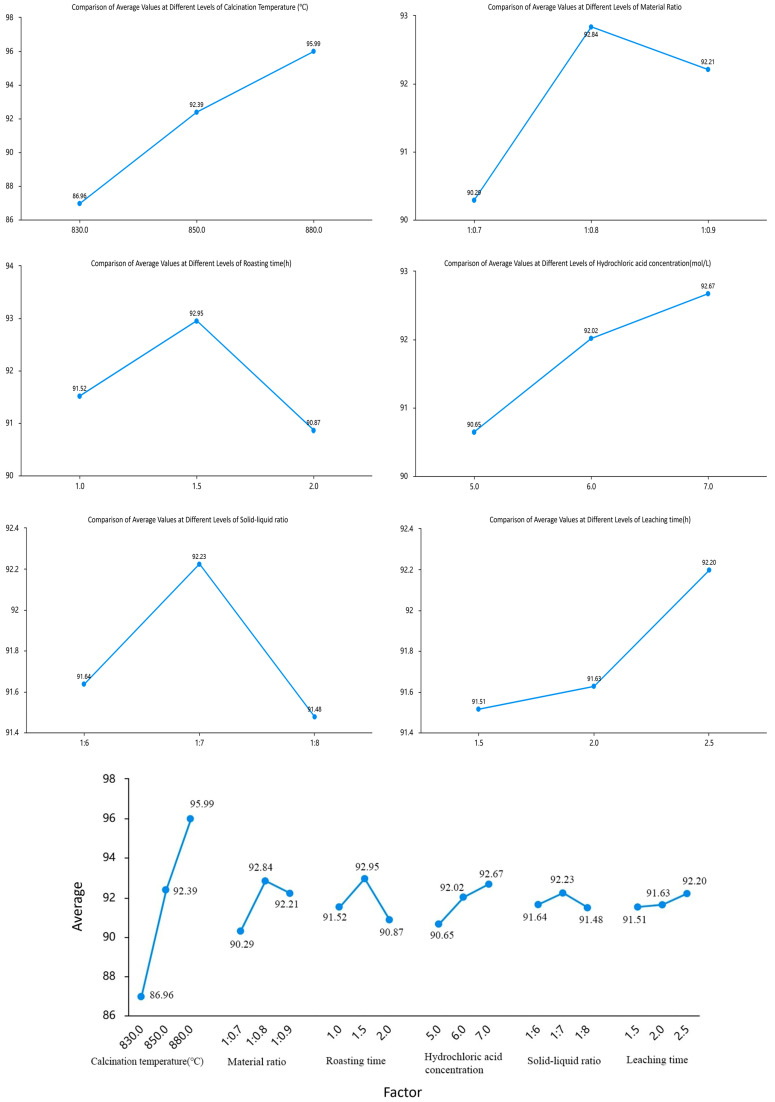
Multi-Factor ANOVA Plot.

Based on the range values (*R*) of each factor in Table 4 and the corresponding range chart of average values at different levels (Figure 13) presenting the results of the multi-factor ANOVA and revealing the significant effects of each factor on the experimental outcomes., the following conclusions can be drawn: The factors are ranked in descending order of importance as follows: calcination temperature, material ratio, calcination time, hydrochloric acid concentration, solid-to-liquid ratio, and leaching time. To demonstrate the relationship between importance and differences, statistical methods were employed to verify the reliability of these differences. Using the extraction amount of aluminum ions as the response variable, a multi-factor ANOVA was conducted with six factors-material ratio, calcination temperature, calcination time, hydrochloric acid concentration, acid leaching time, and solid-to-liquid ratio-as independent variables.

The ANOVA was used to determine whether these factors and their interactions have significant effects on the extraction of aluminum ions. The results of the sum of squares, mean squares (MS), degrees of freedom (df), F-values, and *p*-values for each factor are presented in Table 5. These F-values were compared with the critical F-values at the given significance level (*p* = 0.05).

If the F-value of a factor is greater than the critical F-value, it indicates that the factor has a statistically significant effect on the extraction amount of aluminum ions, suggesting that changes in the levels of this factor will significantly affect the extraction efficiency of aluminum ions. For example, the F-critical value (α = 0.05, numerator degrees of freedom 2, denominator degrees of freedom 4) is 6.94. The F-values and *p*-values are as follows: calcination temperature F = 48.737 (*p* = 0.001), material ratio F = 34.150 (*p* = 0.012), calcination time F = 26.963 (*p* = 0.016), hydrochloric acid concentration F = 22.187 (*p* = 0.019), solid–liquid ratio F = 13.66 (*p* = 0.031), and acid–leaching time F = 9.35 (*p* = 0.043). All passed the significance test (*p* < 0.05), and the F–values of the six factors are greater than 6.94, indicating significant effects.

As shown in Table 5, a multi-factor ANOVA was conducted to investigate the differences in outcomes associated with six factors: calcination temperature, material ratio, calcination time, hydrochloric acid concentration, solid-to-liquid ratio, and leaching time. The model R-squared value was 0.959, indicating that these six controlled factors collectively explained 95.92% of the variation in the results. The analysis revealed that all six factors significantly affected the results (*p* < 0.05). Among them, calcination temperature had the most pronounced effect (*p* = 0.001), while leaching time also had an impact but with a less significant difference compared to the other factors.

## 4. Conclusions

This experiment utilized FA from Hancheng Power Plant as the raw material, achieving the efficient extraction of aluminum by high-temperature calcination with sodium carbonate followed by hydrochloric acid at 100 °C leaching to obtain an aluminum-containing filtrate. The experimental results indicated that when the material ratio of FA to Na_2_CO_3_ was 1:0.7, with a calcination temperature of 880 °C and a calcination time of 1.5 h, XRD analysis revealed that the mullite (3Al_2_O_3_·2SiO_2_) in the FA was essentially completely decomposed and reacted with sodium carbonate to form sodium aluminosilicate (NaAlSiO_4_), sodium metaaluminate (NaAlO_2_), disodium disilicate (Na_2_Si_2_O_5_), nepheline, and sodium metasilicate (Na_2_SiO_3_), with the nepheline phase exhibiting the highest diffraction peak intensity. Under these conditions, after calcination, the fused material was subjected to leaching using 6 mol/L hydrochloric acid at 100 °C with a solid-to-liquid ratio of 1:6 for 2 h, a remarkable enhancement in the aluminum extraction rate is discernible, achieving an aluminum extraction rate of over 97% from the fly ash.

The combined acid-base method for extracting aluminum from FA involves using sodium carbonate as an activating agent, which effectively disrupts the crystal structure of aluminum minerals in the raw fly ash, promoting their transformation into nepheline forms that are more reactive with acids. This approach not only optimizes the efficiency of aluminum extraction but also curtail the production of by-products, thereby substantially alleviating the environmental impact associated with subsequent processing stages. Moreover, this activation method significantly reduces the amount of hydrochloric acid required in the subsequent acid leaching process, thereby decreasing the overall chemical consumption and cost of the process. During the acid leaching stage, hydrochloric acid, as the leaching agent, reacts rapidly with aluminum oxides, allowing for the completion of aluminum extraction in a relatively short time. Compared to other strong acids such as sulfuric acid, hydrochloric acid produces fewer harmful gases during the acid leaching process, reducing the pollution of gas emissions to the environment and the impact on human health. Additionally, the by-products formed from the reaction are stable and do not interfere with the experimental results, ensuring the reliability and reproducibility of the extraction process.

## Figures and Tables

**Table 1:** Mass fractions of fly ash constituents (%).

Component	%
Al_2_O_3_	51.76
SiO_2_	40.43
Fe_2_O_3_	1.72
MgO	0.33
CaO	1.89
Na_2_O	0.28
K_2_O	0.20
TiO_2_	1.41
LOI	1.98

**Table 2:** Table of Controlled Factors and Levels in Orthogonal Experiments.

	Level	Calcination Temperature (°C)	Material Ratio	Roasting Time (h)	Hydrochloric Acid Concentration (mol/L)	Solid-Liquid Ratio	Leaching Time (h)
Factor							
		A	B	C	D	E	F
1		830	1:0.7	1	5	1:6	1.5
2		850	1:0.8	1.5	6	1:7	2
3		880	1:0.9	2	7	1:8	2.5

**Table 3:** Table of L_18_ (3^6^) Orthogonal Experiment and Results.

	Factor							
1	Calcination Temperature (°C)	Material Ratio	Roasting Time (h)	Hydrochloric Acid Concentration (mol/L)	solid-liquid Ratio	Leaching Temperature (°C)	Leaching Time (h)	Response Extraction Rate (%)
NO.	A	B	C	D	E	F	G	
1	830	1:0.7	1.0	5	1:6	100	1.5	83.16
2	830	1:0.7	1.5	6	1:8	100	2.5	87.3
3	830	1:0.8	1.0	7	1:8	100	2.0	89.29
4	830	1:0.8	2.0	5	1:7	100	2.5	87.62
5	830	1:0.9	1.5	7	1:7	100	1.5	88.22
6	830	1:0.9	2.0	6	1:6	100	2.0	86.17
7	850	1:0.7	1.0	7	1:7	100	2.5	92.15
8	850	1:0.7	2.0	5	1:8	100	2.0	86.98
9	850	1:0.8	1.5	6	1:7	100	2.0	94.64
10	850	1:0.8	2.0	7	1:6	100	1.5	93.32
11	850	1:0.9	1.0	6	1:8	100	1.5	93.01
12	850	1:0.9	1.5	5	1:6	100	2.5	94.23
13	880	1:0.7	1.5	7	1:6	100	2.0	97.06
14	880	1:0.7	2.0	6	1:7	100	1.5	95.1
15	880	1:0.8	1.0	6	1:6	100	2.5	95.88
16	880	1:0.8	1.5	5	1:8	100	1.5	96.28
17	880	1:0.9	1.0	5	1:7	100	2.0	95.62
18	880	1:0.9	2.0	7	1:8	100	2.5	96

**Table 4:** Range Analysis Table.

	Factor					
Parameter	A	B	C	D	E	F
*K*_1_	521.76	541.75	549.11	543.89	549.82	549.09
*K*_2_	554.33	557.03	557.73	552.10	553.35	549.76
*K*_3_	575.94	553.25	545.19	556.04	548.86	553.18
r	6.0	6.0	6.0	6.0	6.0	6.0
m	3	3	3	3	3	3
*K*_1,avg_	86.96	90.29	91.52	90.65	91.64	91.51
*K*_2,avg_	92.39	92.84	92.95	92.02	92.23	91.63
*K*_3,avg_	95.99	92.21	90.87	92.67	91.48	92.20
*R*	880	1:0.7	1.5	7	1:6	2.0
	9.03	2.55	2.09	2.03	0.75	0.68

**Table 5:** Results of Multi-Factor ANOVA.

	Sum of Squares	Degrees of Freedom (df)	Mean Square (MS)	F	*p*
Intercept	151,622.396	1	151,622.396	59,603.617	0.000 **
Calcination temperature (°C)	247.959	2	123.980	48.737	0.001 **
Material ratio	21.112	2	10.556	34.150	0.012
Roasting time (h)	13.718	2	6.859	26.963	0.016
Hydrochloric acid Concentration (mol/L)	12.808	2	6.404	22.187	0.019
Solid-liquid ratio	1.863	2	0.932	13.66	0.031
Leaching time (h)	1.604	2	0.802	9.35	0.043
Residual	12.719	5	2.544		

Remark: *R*^2^ = 0.959; * *p* < 0.05 ** *p* < 0.01.

## Data Availability

The data used to support the findings of this study are available from the corresponding author upon request.
